# Evolutionarily Ancient Caspase-9 Sensitizes Immune Effector Coelomocytes to Cadmium-Induced Cell Death in the Sea Cucumber, *Holothuria leucospilota*

**DOI:** 10.3389/fimmu.2022.927880

**Published:** 2022-07-14

**Authors:** Xiaomin Li, Ting Chen, Xiaofen Wu, Zhuobo Li, Xin Zhang, Xiao Jiang, Peng Luo, Chaoqun Hu, Nai-Kei Wong, Chunhua Ren

**Affiliations:** ^1^ CAS Key Laboratory of Tropical Marine Bio-resources and Ecology (LMB), South China Sea Institute of Oceanology, Chinese Academy of Sciences (CAS), Guangzhou, China; ^2^ University of Chinese Academy of Sciences, Beijing, China; ^3^ Southern Marine Science and Engineering Guangdong Laboratory (Guangzhou), Guangzhou, China; ^4^ Institute for Integrative Biology of the Cell, University of Paris-Saclay, Paris, France; ^5^ Clinical Pharmacology Section, Department of Pharmacology, Shantou University of Medical College, Shantou, China; ^6^ Guangdong Provincial Key Laboratory of Infectious Diseases and Molecular Immunopathology, Shantou University Medical College, Shantou, China

**Keywords:** caspase-9, cadmium, reactive oxygen/nitrogen species (ROS/RNS), *Holothuria leucospilota*, invertebrate cell death

## Abstract

Heavy-metal pollution has increasingly jeopardized the habitats of marine organisms including the sea cucumber, a seafloor scavenger vital to seawater bio-decontamination, ocean de-acidification and coral-reef protection. Normal physiology including immune functions of sea cucumbers is toxicologically modulated by marine metal pollutants such as cadmium (Cd). The processes underpinning Cd’s toxic effects on immune systems in the sea cucumber, *Holothuria leucospilota*, are still poorly understood. To this end, we cloned and characterized a full-length caspase-9 (*Hl-CASP9*) cDNA in the sea cucumber, *Holothuria leucospilota*. *Hl-CASP9* mRNA levels evolved dynamically during embryonic development. Coelomocytes, a type of phagocytic immune effectors central to *H. leucospilota* immunity, were found to express *Hl-CASP9* mRNA most abundantly. Hl-CASP9 protein structurally resembles caspases-2 and -9 in both invertebrate and vertebrate species, comprising a CARD domain and a CASc domain. Remarkably, *Hl-CASP9* was transcriptionally sensitive to abiotic oxidative stress inducers including hydrogen peroxide (H_2_O_2_), nitric oxide (^•^NO) and cadmium (Cd), but insensitive to immunostimulants including lipopolysaccharide (LPS), and poly(I:C). Overexpression of *Hl-CASP9* augmented mitochondria-dependent apoptosis in HEK293T cells, while knock-down of *Hl-CASP9* blunted Cd-induced coelomocyte apoptosis *in vivo*. Overall, we illustrate that an evolutionarily ancient caspase-9-dependent pathway exists to sensitize coelomocytes to premature cell death precipitated by heavy metal pollutants, with important implications for negative modulation of organismal immune response in marine invertebrates.

## Introduction

Sea cucumbers (Echinodermata: *Holothuroidea*) constitute a major scavenging component integral to many marine ecosystems such as coral reefs, kelp grasslands and mangroves (1). Among these, *Holothuria leucospilota* is a tropical species endemic to the Indo-Pacific region, which dwells amidst coral reefs, mud or sand (2, 3). The sea cucumber typically feeds on sediments rich in organic carbons. It possesses a powerful digestive tract that efficiently degrades organic carbons, concomitantly reducing biocontamination and promoting local pH balance of seawater (4, 5). Thus, *H. leucospilota* serves as a seabed scavenger vital to the health and biodiversity of nearshore marine ecosystems, and has been prized for their roles in the restoration of injured coral reefs. However, the sea cucumber lacks an adaptive immune system as in other marine invertebrates; they rely overwhelmingly on innate immunity including humoral and cellular responses (6). In particular, the phagocytic coelomocytes (literally, cells derived from the coelom, a fluid-filled cavity occupying the space between the alimentary canal and body walls) have been recognized as the primary effector cells in the immune system of echinoderms (e.g., the sea cucumber), with indispensable roles in cell agglutination, encapsulation, phagocytosis and cell death (7, 8).

Within the increasingly volatile marine environments, physicochemical properties of seawater (e.g., pH, composition, etc.) can be adversely affected by anthropogenic pollutants from industrial discharges. Among the most deleterious pollutants are heavy metals, namely, cadmium (Cd), mercury, lead, and arsenic (9). The presence of Cd in ocean waters, for example, exerts global survival pressure on marine life, especially for species dwelling in the delicate coral-reef ecosystems, which ultimately leads to a profound loss of marine biodiversity (10, 11). In addition, Cd accumulates in tissues of marine organisms and could well cause harm to human health by entering or re-entering the food chain (12). Among marine benthos, echinoderms represent a simple but important model for assessing the effects of specific environmental pressure on biological growth and vitality (9). As heavy metals are generally toxic and difficult to remove, their accumulation may lead to acute or chronic oxidative stress in aquatic animals, which is accompanied by excessive ROS production, protein dysfunction, lipid peroxidation and DNA oxidation (12, 13).

As a key protein family involved with apoptosis, cysteine-dependent aspartate-directed proteases (caspases) are a family of cysteine proteases ubiquitously found in vertebrate and invertebrate species including echinoderms (14). Typically, apoptotic response is mediated through either an extrinsic or an intrinsic pathway. The extrinsic pathway is triggered by binding of extracellular ligands to death receptors on the cell membrane, whereas the intrinsic pathway is mainly turned on by different mitochondrial stressors, leading to the cytoplasmic release of mitochondrial cytochrome c, which then binds caspase-9 and apoptotic protease activating factor 1 (Apaf-1) to form an apoptosome (15). As an initiator caspase, caspase-9 is prerequisite for mitochondrial morphological changes and ROS production (16). Interestingly, despite its relevance to cell death, caspase-9 also plays pivotal roles in tissue differentiation (e.g., in muscles) in non-apoptotic signaling (17).

In recent years, there has been growing interest in the biology of apoptotic caspases in ecologically important echinoderms, including sea urchin (*Paracentrotus lividus*), sea cucumber (*H. leucospilota* and *Apostichopus japonicus*) and starfish (*Asterina pectinifera*) (18–22) (See a summary on this in **Table 1**). For example, exposure of *P. lividus* embryos to Cd triggered autophagy and concomitant apoptosis as assessed by TUNEL and cleaved caspase-3 (30). In previous studies on *H. leucospilota* by our group, *HLcaspase-6* was shown to be an effector caspase in apoptosis (21), while *HLcaspase-8* was found to operate in a receptor-related apoptotic pathway (22). Both *HLcaspase-6* and *HLcaspase-8* were transcriptionally up-regulated by immunostimulants such as lipopolysaccharide (LPS) or polyinosinic-polycytidylic acid [poly(I:C)] in *H. leucospilota* coelomocytes *in vitro*. Furthermore, both *caspase-9* and *Apaf-1* are present in the genome of *Strongylocentrotus purpuratus* (sea urchin), lending support to a conserved mechanism governing intrinsic apoptosis within deuterostomes (34). However, in echinoderms as in other marine invertebrates, the regulatory machinery for caspase-dependent pathways in Cd-induced apoptosis remains obscure.

**Table 1:** Recent works on marine invertebrate caspases in immunological or toxicological contexts.

Caspase	Organism	Model	Treatment	Effects on caspase expression or activity	Ref.
CASP9	*H. leucospilota*	*In vitro* in coelomocytes; *in vivo* by knock-down of CASP9	LPS, poly(I:C), *V. harveyi*; H_2_O_2_, ^•^NO, Cd	No effects from immunostimulants; stimulatory with oxidative stressors; increased apoptosis	This study
CASP1	*A. japonicus*	*In vitro*	*V. splendidus*, LPS	Stimulatory	(23)
CASP1	*C. gigas*	*In vitro*	LPS	Stimulatory	(24)
CASP1	*M. japonicas*	*In vivo*	White spot syndrome virus	Stimulatory	(25)
CASP3	*A. japonicus*	*In vitro*	LPS	Stimulatory	(26)
CASP3	*C. hongkongensis*	*In vitro*	*V. alginolyticus*, *S. haemolyticus*	Stimulatory	(27)
CASP3, CASP9	*C. gigas*	*In vitro*	CO_2_	Stimulatory in the short term	(28)
CASP3	*P. monodon*	*In vivo*, inhibition of CASP3	Yellow head virus	CASP3 activity is blocked	(29)
CASP3, CASP8	*P. lividus*	*In vitro*	Diatom-derived oxylipins	Stimulatory	(18)
cleaved CASP3	*P. lividus*	*In vitro* in embryos	Cd	Stimulatory	(30)
CASP3, CASP9	*S. japonicus*	*In vitro*	Ultraviolet light	Stimulatory	(31)
CASP3/7	*S. paramamosain*	*In vitro*	*V. parahaemolyticus*, *V. alginolyticus*, LPS	Stimulatory	(32)
CASP6	*H. leucospilota*	*In vitro*	LPS, poly(I:C)	Stimulatory	(21)
CASP8	*H. leucospilota*	*In vitro*	LPS, poly(I:C), TNF-α	Stimulatory	(22)
CARDCP	*C. gigas*	*In vitro*	LPS, *V. splendidus*	Stimulatory	(33)

Species: Holothuria leucospilota (sea cucumber); Apostichopus japonicus (sea cucumber); Crassostrea gigas (oyster); Marsupenaeus japonicas (shrimp); Crassostrea hongkongensis (oyster); Penaeus monodon (shrimp); Paracentrotus lividus (sea urchin); Stichopus japonicus (sea cucumber); Scylla paramamosain (crab). Gram-negative marine pathogens: Vibrio harveyi; Vibrio splendidus; Vibrio parahaemolyticus; Vibrio alginolyticus. Gram-positive marine pathogens: Staphylococcus haemolyticus.

In order to clarify the physiological roles of caspase-9 in echinoderms, we first cloned and characterized caspase-9 cDNA from tropical sea cucumber, *H. leucospilota*, an ecologically relevant and commercially valuable aquaculture species in southern China (35). To scrutinize the function of *H. leucospilota* caspase-9 (*Hl-CASP9*), we analyzed mRNA distribution profiles across developmental stages and various adult tissues of *H. leucospilota*, and compared transcriptional expression profiles following pharmacological challenges of immunostimulants and oxidative stressors including Cd and chemical donors of ROS/RNS (reactive oxygen/nitrogen species). Furthermore, the functional relevance of *Hl-CASP9* to mitochondrial stress and apoptosis in response to Cd was also examined. Our current work thus provides fresh insights into the functional significance of sea cucumber caspase-9 in relation to environmental perturbations such as heavy metal contaminants and microbial infections, which should help stimulate interest in regulatory mechanisms underlying cell death and stress adaptation in marine invertebrates including echinoderms.

## Materials and Methods

### Animals and Tissue Collection

Healthy adult individuals of sea cucumbers (*H. leucospilota*) weighing 200 ± 10 g were obtained from Daya Bay (Shenzhen, China) and temporarily reared in a seawater aquarium with filtrated and aerated seawater with salinity of 35‰ and temperature of 32°C for a week before experiments. After being placed on ice for 30 min, sea cucumbers were dissected and the tissues were collected and frozen rapidly in liquid nitrogen for storage at −80°C, until RNA extraction. Meanwhile, coelomic fluids were filtered by 100 µm sterile nylon mesh and centrifuged immediately at 1,000 × g for 10 min at 4°C to harvest coelomocytes. Then, the harvested coelomocytes were then kept in 1 mL TRIzol reagent (Invitrogen, USA) at −80°C. All animal experiments have been approved by the Ethics Committees of the South China Sea Institute of Oceanology, Chinese Academy of Sciences, and were conducted in accordance with guidelines thereof.

### Molecular Cloning of *Hl-CASP9* cDNA

Total RNA from coelomocytes of *H. leucospilota* was extracted by using TRIzol reagent and treated with RNase-free recombinant DNase I (Takara, Japan). First-strand cDNA was synthesized by using the PrimeScript™ II 1^st^ Strand cDNA Synthesis Kit (Takara). Based on a transcriptomic library of *H. leucospilota* coelomocytes previously constructed by our lab, a partial sequence for the *Hl-CASP9* homologue was verified with gene-specific primers *Hl-CASP9*-F and *Hl-CASP9*-R (**Table S1**). To obtain corresponding full-length cDNA sequences, the 3′- and 5′-rapid amplification of cDNA ends (RACE) was performed by using 3′ Full Race Core Set Ver. 2.0 and 5′ Full Race Kit (Takara) with gene-specific primers (3′ RACE1/3′ RACE2 and 5′ RACE1/5′ RACE2, see **Table 1**), respectively. Amplification conditions for verification of partial sequences and RACE were set as follows: 94°C for 60 s, 30 cycles of 98°C for 10 s, 55°C for 15 s and 68°C for 60 s. pMD™ 19-T vector (Takara) and DH5*α* competent cells (Takara) were used for subcloning of the amplicons. Three positive clones for each amplicon were subsequently sequenced.

### Bioinformatics Analysis

Open reading frame (ORF) and amino acids sequences were predicted by using ORF Finder (https://www.ncbi.nlm.nih.gov/orffinder/). Structural domains of Hl-CASP9 were predicted by using the SMART (http://smart.embl-heidelberg.de/), ScanProsite (http://prosite.expasy.org/) and BLAST (http://blast.ncbi.nlm.nih.gov) programs. Molecular weight (M.W.) and theoretical isoelectric point were calculated according to deduced amino acids by ProtParam (http://web.expasy.org/protparam/). Alignment for amino acids sequences among various species was performed with the Clustal Omega program (http://www.ebi.ac.uk/Tools/msa/clustalo/) and presented by using the Jalview program (http://www.jalview.org/). Phylogenetic trees were constructed based on amino acid differences (*p*-distance) according to the neighbor-joining method (pairwise deletion) with 1,000 bootstrap replicates by using MEGA X. Three-dimensional (3-D) models were deduced with the I-TASSER server (http://zhanglab.ccmb.med.umich.edu/I-TASSER/) and visualized by the VDM program (http://www.ks.uiuc.edu/Research/vmd/).

### Spatial Expression Patterns of *Hl-CASP9* mRNA in Different Developmental Stages and Adult Tissues

Morphology of *H. leucospilota* embryos and larvae was studied by using an optical microscope, as described previously by our group (35). Samples were collected at different developmental stages including: fertilized egg, 2-cell, 4-cell, 8-cell, 16-cell, morula, blastula, rotated-blastula, early-gastrula, late-gastrula, early-auricularia, mid-auricularia, auricularia, doliolaria, pentactula, and juvenile stages. Tissue distribution of *Hl-CASP9* mRNA was quantitatively detected in three adult individuals. The selected tissues include coelomocytes, intestine, body wall, respiratory tree, rete mirabile, transverse vessel, Polian vesicles, muscle, oesophagus, Cuvierian tubules and gonads. Total RNA was extracted with TRIzol reagent and digested with gDNA Eraser (Takara) and reverse transcription by using the PrimeScript™ RT reagent Kit (Takara) for quantitative PCR (qPCR). Specific primers Q*Hl-CASP9*-F and Q*Hl-CASP9*-R (**Table S1**) were designed based on the obtained *Hl-CASP9* cDNA sequences. qPCR reactions were performed by using SYBR Premix Ex Taq™ II (Takara) in a final volume of 20 μL, with the conditions of 40 cycles of 95°C for 5 s and 60°C for 30 s. In this experiment, *Hl-β-actin* was used as an internal control to verify qPCR results with reference to previous modifications (22).

### Expression of *Hl-CASP9* mRNA in Sea Cucumber Primary Coelomocytes Following Immune or Oxidative Challenges

Coelomocytes were chosen as a cell model for investigating transcriptional responses of *Hl-CASP9* following challenges of immunostimulants or oxidative stressors. Primary sea cucumber coelomocytes were prepared as previously described (36). After culture at 28°C for 18 h in Leiboviz’s L-15 culture medium (with 0.39 M NaCl) (Invitrogen), coelomocytes were challenged with immunostimulants including LPS (10 μg/mL), poly(I:C) (10 μg/mL) and heat-inactivated *V. harveyi* (10^7^ CFU/mL), an important pathogen of many aquatic animals, or alternatively, with oxidative stress inducers including H_2_O_2_ (hydrogen peroxide; 2 μM), NOC-18 (a nitric oxide donor; 1μM) and CdCl_2_ (cadmium chloride; 20 μM), respectively. After these treatments for 0, 3, 6, 12 and 24 h, coelomocytes were harvested and lysed in TRIzol reagent, followed by procedures for RNA extraction and, first-strand cDNA synthesis. Quantitative changes in mRNA expressions of *Hl-CASP9* after immune and oxidative challenges were analyzed quantitatively by qPCR assays, as described above.

### Construction of Recombinant Plasmid pcDNA3.1(+)/*Hl-CASP9*

Coding region of *Hl-CASP9* was amplified by PCR by using the gene-specific primers P*Hl-CASP9*-F and P*Hl-CASP9*-R (**Table S1**), with restriction enzyme sites for *Bam*H I and *Xho* I. PCR products and the pcDNA3.1(+) vector (Invitrogen) were digested with *Bam*H I (Takara) and *Xho* I (Takara), and ligated by means of T4 DNA ligase (Fermentas, USA) at 16°C for 1 h and then used to transform DH5α competent cells. Accuracy of the constructed plasmid was confirmed by double-enzyme (*Bam*H I and *Xho* I) digestion and sequencing. A positive clone was picked and cultured in lysogen broth (LB) containing ampicillin (100 μg/mL) at 37°C with shaking at 220 rpm overnight. The amplified pcDNA3.1(+)/*Hl-CASP9* recombinant plasmid was purified by using the Plasmid MiniPrep DNA Kit (Axygen, USA).

### HEK293T Cell Culture and Transfection

Before transfection, HEK293T cells were seeded in a 6-well plate and cultured in Dulbecco’s Modified Eagle Medium (Hyclone, USA) containing 10% fetal calf serum (FCS), penicillin (100 μg/mL) and streptomycin (100 μg/mL) at 37°C with 5% CO_2_ for one day. Afterwards, HEK293T cells were transfected with 3 μg pcDNA3.1(+)/*Hl-CASP9* recombinant plasmid by using 3 μL lipofectamine 2000 (Invitrogen) according to the manufacturer’s instructions. By using the same methods, HEK293T cells were transfected with an empty pcDNA3.1(+) vector as a control.

### Detection of Apoptosis in *Hl-CASP9* Overexpressing HEK293T Cells

HEK293T cells were incubated in the presence or absence of CCCP (carbonyl cyanide 3-chlorophenylhydrazone; 100 μM), a protonophore which uncouples oxidative phosphorylation in mitochondria, at 37°C for 30 min. Then, apoptosis was detected by an Annexin V-FITC/PI cell apoptosis detection kit (Solarbio, China). Cells were collected and washed twice with pre-cooled PBS (pH 7.4) and resuspended in 1×binding buffer, with a final cell suspension density of 1×10^6^ cells/mL. Subsequently, 100 μL cell suspension mixed with 5 μL Annexin V-FITC and 5 μL propyl iodide (PI) was incubated in dark for 10 min at room temperature. Then, 400 μL of 1 × binding buffer was added prior to detection. Finally, cell apoptosis proportions were measured by flow cytometry (Beckman Coulter, USA) within 1 h. Annexin V-FITC was used to stain phospholipid serine on the cell membrane at the early stage of apoptosis, wherein FITC signals indicate the rate of early apoptosis. PI penetrates membranes of damaged cells, such as late apoptotic cells or necrotic cells, and binds nuclear DNA to give red staining. Cells doubly stained by PI and Annexin V-FITC are considered late apoptotic cells or necrotic cells.

### Cellular ROS Detection in Primary Coelomocytes

Primary coelomocytes (1×10^6^ cells/mL, survival rate > 95%) were seeded into 6-well plates pre-coated with poly D-lysine and grown overnight in Leiboviz’s L-15 culture medium (with 0.39 M NaCl). After treatment with different concentrations of CdCl_2_ (0, 5, 10 or 20 μM) for 3 h, the medium was removed. Then, 1 μM dichloro-dihydro-fluorescein diacetate (DCFH-DA) (Biosharp, China) diluted in serum-free medium was added. After 30-min incubation, coelomocytes were washed twice in PBS (with 0.53 M NaCl), followed by observation under a fluorescent microscope (Zeiss, Germany). Fluorescence intensity quantification was done with ImageJ.

### Knock-Down of *Hl-CASP9* by RNA Interference (RNAi) in Sea Cucumbers *In Vivo*

Double-stranded RNA (dsRNA) targeting the *Hl-CASP9* gene was synthesized by using the T7 RiboMAX™ Express RNAi System (Promega, USA) according to the manufacturer’s instructions. Similarly, a GFP cDNA fragment was amplified as the negative control. Specific primers used for the synthesis of dsRNA, as shown in **Table S1**. Concentration of synthesized dsRNA was verified by NanoDrop 2000 spectrophotometer (Thermo Scientific, USA), which was then diluted to 600 ng/μL in 150 mM RNase-free saline solution (RFSS). For *in vivo* dsRNA interference, nine healthy individuals of *H. leucospilota* (each weighing about 100 g) were randomly assigned into three groups (n = three individuals per group). The experimental group was injected with 100 μL dsCASP9, while the control group was injected with 100 μL RFSS and the negative control was injected with an equivalent amount of dsGFP. Following RNAi for 48 h, all sea cucumbers were dissected on the ice and their coelomocytes were isolated. Thereafter, a sample of coelomocytes was taken to assess interference efficiency of RNAi experiment by qPCR, with specific primers and reaction conditions identical to those listed in section 2.4.

### Detection of Mitochondrial Superoxide Formation in Primary Coelomocytes

Primary coelomocytes (1×10^6^ cells/mL) from different groups (sea cucumbers injected with RFSS, dsGFP or dsCASP9) were plated into 3.5-cm glass-bottomed confocal dishes pre-coated with poly D-lysine, followed by incubation at 28°C for 18 h and subsequent treatment with CdCl_2_ (20 μM) for 3 h. Prior to imaging experiments, coelomocytes were loaded with the mitochondrial superoxide probe MitoSOX (2.5 μM; Invitrogen) for 30 min and then washed with HBSS (with 0.53 M NaCl) immediately before imaging. Images were obtained with a confocal fluorescence microscope (Leica, Germany). Fluorescence intensity quantification was done with ImageJ.

### Mitochondrial Membrane Potential Detection in Primary Coelomocytes

Mitochondrial membrane potential (Δ*ψ*_m_) was measured by the commercial membrane potential probe JC-1 (2.5 μM; Invitrogen). Briefly, primary coelomocytes (1×10^6^ cells/mL) from different groups (sea cucumbers injected with RFSS, dsGFP or dsCASP9) were seeded into 6-well plates pre-coated with poly D-lysine and grown in Leiboviz’s L-15 culture medium (with 0.39 M NaCl) overnight. Then, coelomocytes were treated with CdCl_2_ (20 μM; 3 h) or CCCP (100 μM; 30 min), respectively. After removing culture medium, coelomocytes were incubated with JC-1 for 20 min in dark and then washed twice with PBS (with 0.53 M NaCl) before acquisition. Δ*ψ*m in coelomocytes from different groups was detected by flow cytometry (Beckman Coulter).

### Coelomocyte Apoptosis Assay by Flow Cytometry Following RNAi

After RNAi for 48 h, primary coelomocytes from different groups (sea cucumbers injected with RFSS, dsGFP or dsCASP9) were harvested and cultured overnight in Leiboviz’s L-15 cell culture medium (with 0.39 M NaCl), and then exposed to oxidative stress inducers including H_2_O_2_ (2 μM), NOC-18 (1 μM) and CdCl_2_ (20 μM). After 24 h, the *H. leucospilota* coelomocytes were resuspended by proper pipetting and the coelomocytes proportions of undergoing apoptosis was detected by an Annexin V-FITC/PI cell apoptosis detection kit (Solarbio) and determined by flow cytometry (Beckman Coulter) as described above.

### Data Transformation and Statistical Analysis

All data were analyzed by using one-way ANOVA and presented as mean ± SEM (*n* = 3), followed by Fisher’s least significant difference (LSD) test with SPSS 22.0 (IBM Software, USA). Statistical significance was determined at *p* > 0.05 (i.e., n.s.), *p < 0.05, **p < 0.01 or ***p < 0.001.

## Results

### Molecular Cloning and Sequence Analysis of *Hl-CASP9*

**Figure 1:**
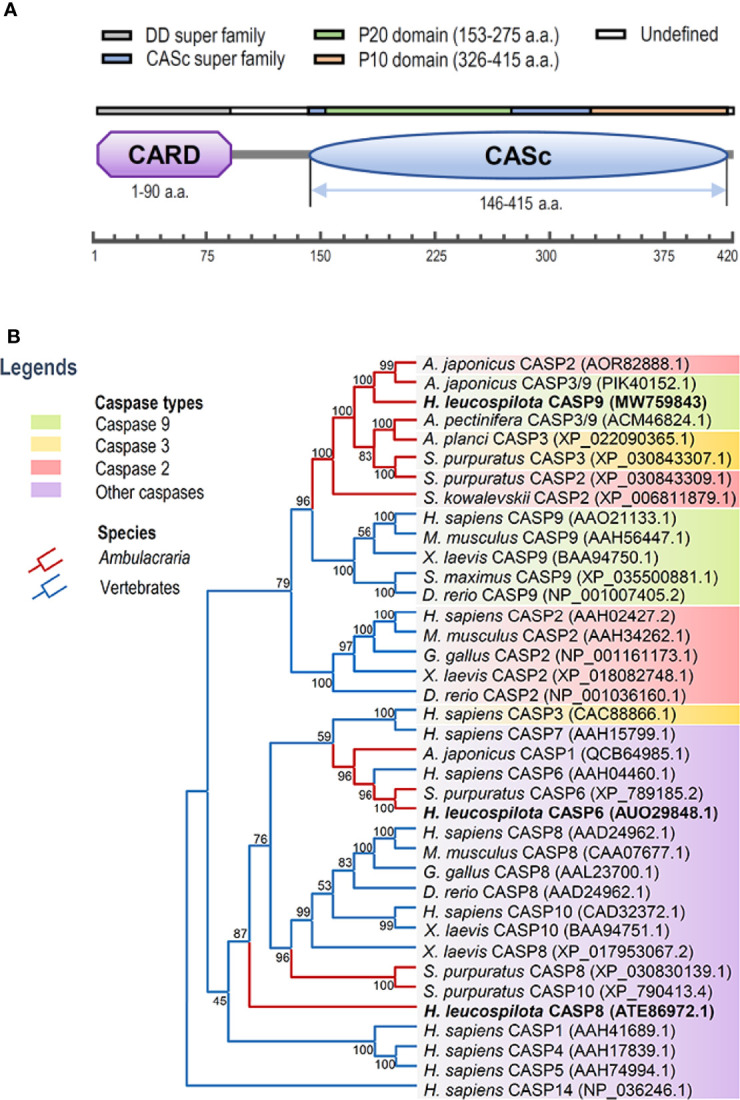
Functional domains of *Hl-CASP9* and its evolutionary relationships with other caspases. **(A)** Structural domains of *Hl-CASP9* predicted by using the SMART and ScanProsite programs. **(B)** Phylogenetic analysis of caspases among various species by using Neighbor-Joining method with bootstrap value of 1,000.

In this study, full-length cDNA sequence of caspase-9 was obtained from *H. leucospilota* by using 3’-/5’-RACE approaches, which has been deposited in GenBank under the accession number MW759843. The *Hl-CASP9* cDNA is 1,841 bp in length, encompassing a 5’-untranslated region (UTR) of 180 bp, a 3’-UTR of 398 bp and an ORF of 1,263 bp encoding a protein of 420 a.a. (**Figure S1A**) with a deduced molecular weight of 47.25 kDa and a predicted isoelectric point of 6.44. Based on analysis with the SMART program, a caspase recruitment domain (CARD, residues 1-90) at the *N*-terminus, and a CASc domain (residues 146-415) at the *C*-terminus were predicted in the amino acid sequence of *Hl-CASP9* (**Figure 1A**). Within its CASc domain, two active sites (His228 and Cys271), a substrate pocket (containing 12 conserved amino acids), a proteolytic cleavage site (containing 2 conserved amino acids) and a dimer interface (containing 15 conserved amino acids) were determined with the Blast program in NCBI (**Figure S1B**). By using ScanProsite prediction, the classical caspase subunits P20 (residues 153-275) and P10 (residues 326-415) within the CASc domain were identified. Moreover, the caspase family histidine active site motif (^215^HSNFDCVALAILTHG^229^) and the cysteine active site motif (^262^KPKIIILQACRG^273^) were also found in the P20 subunit (**Figure S1A**).

### Phylogenetic and Structural Analyses

**Figure 2:**
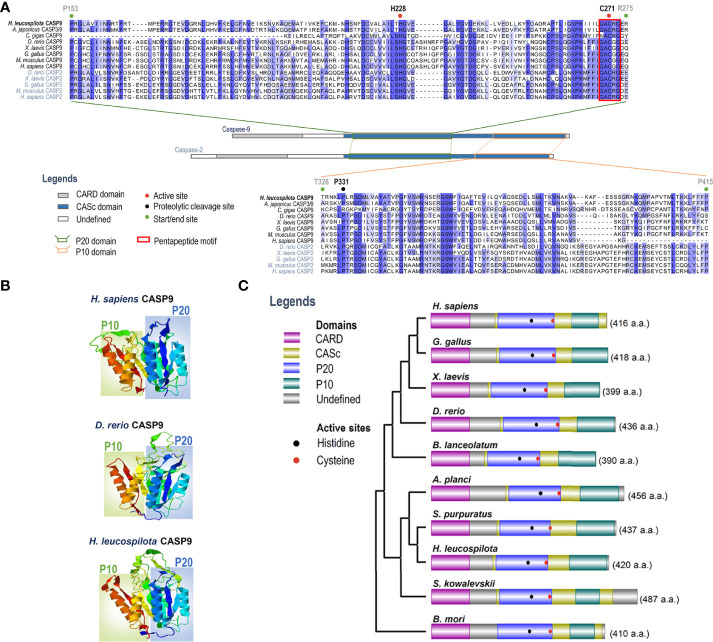
Analysis on amino acid sequence alignment, and organization of domains and 3-D structures of caspase-9. **(A)** Amino acid sequence alignment of caspase-2 and caspase-9 in various species. Conserved amino acid residues are boxed in dark blue, and similarities in amino acid residues are highlighted in light blue. **(B)** Comparison on 3-D structures of CASc domains in caspase-9 among three species. Positions of the P20 and P10 subunits are boxed. **(C)** Protein structural organization of caspase-9 among various species.

To clarify evolutionary relationships of caspases within deuterostomes, a phylogenetic tree was constructed. As shown in **Figure 1B**, caspase-9 and caspase-2 were clustered into one main branch, whereas other caspases were clustered into another main branch of the dendrogram. In the branch of caspase-2/9, Ambulacraria (represented by the phyla Echinodermata and Hemichordata) caspase-2/9 and vertebrate caspase-9 were clustered into one clade, whereas vertebrate caspase-2 was grouped into another clade. In addition, our newly identified *Hl-CASP9* was highly similar in sequence to the sea cucumber *A. japonicus* caspase-2 and caspase-3/9, which have been named as *Ajcaspase-2* and *Ajcaspase-3/9* (19), respectively. Multiple alignments of amino acid sequences show that Hl-CASP9 proteins in different species share considerable consensus sequences among echinoderms and vertebrates. The catalytic CASc domain of caspase-9 and caspase-2 is remarkably conserved, especially in the P20 and P10 subunits. Unexpectedly, the *Hl-CASP9* pentapeptide “QACRG” active sites were located within the P20 domain (**Figure 2A**), instead of the conserved pentapeptide “QACGG” found in the caspase-9 of vertebrates (37). In 3-D modeling, for caspase-9 CASc domains constructed with the amino acid sequences from human, zebrafish and sea cucumber *H. leucospilota* (**Figure 2B**), inter-species structural similarities in the P20 and P10 were confirmed. Likewise, the CARD domain and nestled active sites in the caspase-9 proteins are conserved (**Figure 2C**).

### Expression Patterns of *Hl-CASP9* mRNA Across Developmental Stages and Adult Tissues

**Figure 3:**
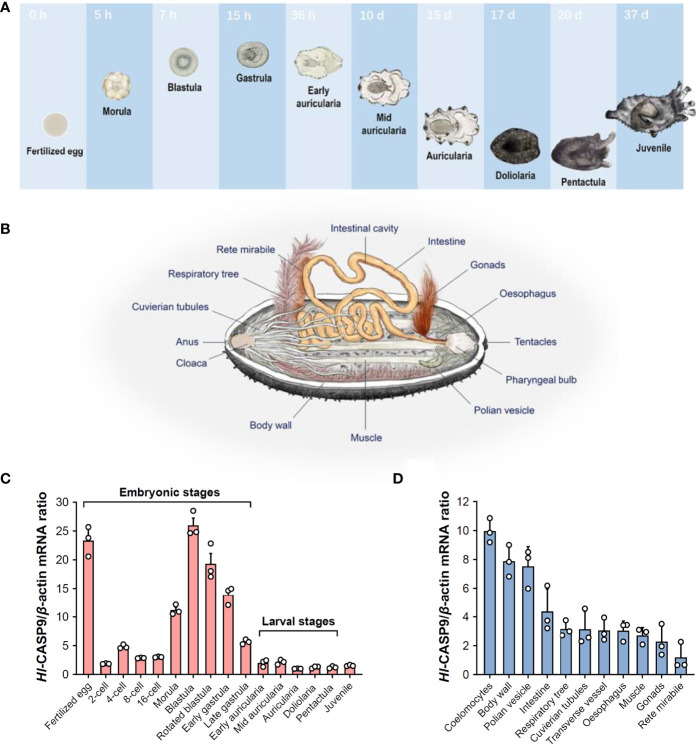
Spatial and temporal expression patterns of *Hl-CASP9* mRNA in developing embryos and adult tissues. **(A)** Typical embryonic and larval development of *H. leucospilota*. Numbers indicate time lapsed post-fertilization. **(B)** Anatomy of *H. leucospilota*. **(C)** *Hl-CASP9* mRNA expression profiles in developing embryos and larvae of *H. leucospilota*. **(D)** *Hl-CASP9* mRNA expression profiles in different adult tissues of *H. leucospilota*.

To characterize spatial and temporal expression patterns of *Hl-CASP9*, its transcript profiles in developing *H. leucospilota* embryos and larvae, as well as those in eleven selected adult tissues, were analyzed by qPCR. The representative developmental stages and anatomical architecture of the sea cucumber *H. leucospilota* are as shown in **Figures 3A, B**, respectively. *Hl-CASP9* expression was found to be dynamic in all embryonic stages and fluctuated significantly, whereas *Hl-CASP9* levels stayed relatively low in larval stages (**Figure 3C**). Specifically, *Hl-CASP9* mRNA levels were high at the fertilized egg stage, but fell sharply at the early cleavage stage. As complexity of the embryos evolved, expression levels of *Hl-CASP9* mRNA rose progressively, reaching a peak at the blastula stage, before subsiding again slowly in subsequent stages. Based on these observations, it could be speculated that *Hl-CASP9* is implicated in some apoptosis- or caspase activity-dependent embryonic processes of *H. leucospilota*. As shown in **Figure 3D**, *Hl-CASP9* transcripts were amply expressed in most adult tissues examined. Compared with the expression of rete mirabile, where the lowest expression levels were detected, the highest expression of *Hl-CASP9* was found in the coelomocytes, followed by the body wall, muscle, oesophagus, Polian vesicles, intestine, gonads, respiratory tree, Cuvierian tubules and transverse vessel.

### Transcriptional Response of *Hl-CASP9* Following Treatment of Immunostimulants or Oxidative Stress Inducers

**Figure 4:**
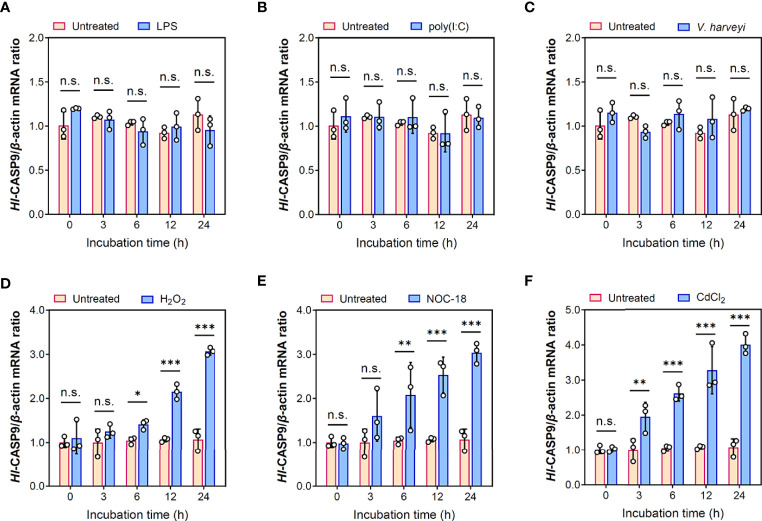
Transcriptional expression patterns of *Hl-CASP9* following challenges of immunostimulants or oxidative stress inducers. **(A)** Time-course study on the expression of *Hl-CASP9* mRNA in coelomocytes treated with LPS (10 μg/mL). **(B)** Time-course study on the expression of *Hl-CASP9* mRNA in coelomocytes treated with poly(I:C) (10 μg/mL). **(C)** Time-course study on the expression of *Hl-CASP9* mRNA in coelomocytes treated with inactivated *V. harveyi* (10^7^ cells/mL). **(D)** Time-course study on the expression of *Hl-CASP9* mRNA in coelomocytes treated with H_2_O_2_ (2 μM). **(E)** Time-course study on the expression of *Hl-CASP9* mRNA in coelomocytes treated with NOC-18 (1 μM). **(F)** Time-course study on the expression of *Hl-CASP9* mRNA in coelomocytes treated with CdCl_2_ (20 μM).

Dynamic changes in *Hl-CASP9* transcription in primary coelomocytes following challenges with immunostimulants LPS, poly(I:C) and heat-inactivated *V. harveyi* are as shown in **Figures 4A-C**. In this case, no significant changes were found in the expression of *Hl-CASP9* mRNA from 0 to 24 h post-challenge. In contrast, *Hl-CASP9* mRNA expression in primary coelomocytes were significantly upregulated under oxidative stressors including H_2_O_2_, NOC-18 and CdCl_2_ (**Figures 4D-F**). In response to H_2_O_2_ treatment, the expression of *Hl-CASP9* was initially upregulated at 6 h and reach a peak of 2.87 folds (*p* < 0.001) relative to the untreated baseline at 24 h post-challenge. When treated with NOC-18, a well-established chemical donor of nitric oxide (^•^NO) (38), the expression of *Hl-CASP9* was significantly elevated at 6 h and reached a peak of 2.84-fold (*p* < 0.001) increase relative to the basal levels at 24 h post-challenge. In the case of Cd, *Hl-CASP9* expression was strongly stimulated: a 1.94-fold (*p* < 0.01) increase at 3 h following CdCl_2_ treatment, and a peak of 3.74-fold (*p* < 0.001) increase at 24 h. Taken together, these results suggest that *Hl-CASP9* mRNA expression seems differentially regulated in response to oxidative stressors and immunostimulants.

### Detection of ROS Formation in Coelomocytes Under Cd Stress

**Figure 5:**
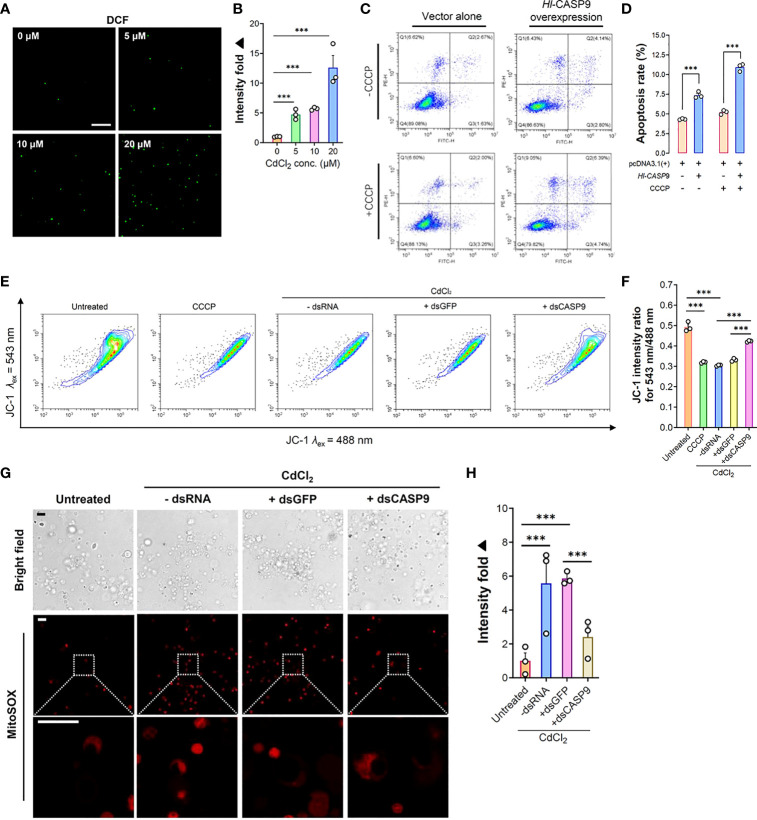
Formation of cellular ROS and associated mitochondrial stress in CdCl_2_-treated coelomocytes. **(A)** *In vitro* dose-dependent effects of CdCl_2_ (at 3 h) on ROS formation levels in primary coelomocytes as detected by the fluorescent DCF. Scale bar: 50 μm. **(B)** Quantification of DCF fluorescence intensities as reported in **(A)**. **(C)** Effects of *Hl-CASP9* overexpression on apoptosis in HEK293T cells, as analyzed by flow cytometry. HEK293T cells were treated with or without CCCP (100 μM). Comparisons are made between group with vector alone (HEK293T cells transfected with pcDNA3.1(+) blank plasmid) and group overexpressing *Hl-CASP9* (HEK293T cells transfected with pcDNA3.1(+)/*Hl-CASP9* recombinant plasmid). **(D)** Comparison on average apoptosis rates in HEK293T cells treated with or without CCCP in different groups. **(E)** Changes in mitochondrial membrane potential (Δ*ψ*m) in CdCl_2_-treated primary coelomocytes with or without dsRNA (including dsGFP and dsCASP9) as assessed by JC-1 (2.5 μM) in flow cytometry. For comparison, coelomocytes were treated with or without CCCP (100 μM) or CdCl_2_ (20 μM). **(F)** Changes of Δ*ψ*m in coelomocytes in response to CdCl_2_ were quantified by JC-1 intensity ratio for *λ*_ex_ 543 nm/488 nm. **(G)** *In vitro* effects of CdCl_2_ (20 μM) on mitochondrial superoxide formation in primary coelomocytes with or without dsRNA (including dsGFP and dsCASP9), as visualized by MitoSOX (2.5 μM) in confocal imaging. Scale bar: 15 μm. **(H)** Quantification of MitoSOX fluorescence intensities in coelomocytes. Data were analyzed by using ImageJ.

ROS formation and its associated oxidative damage are recognized as hallmarks of heavy metals-induced cytotoxicity (12). To illustrate the relationship between ROS production and CdCl_2_ exposure in sea cucumber coelomocytes, intracellular ROS levels in coelomocytes were detected by means of the non-fluorescent probe DCFH-DA, which is switched on in a fluorescent state as DCF on reaction with ROS (39). As shown in **Figure 5A**, ROS formation in coelomocytes was dramatically elevated upon CdCl_2_. At 3 h post-Cd challenge, ROS levels were augmented by 4.73 folds (*p* < 0.001) relative to the untreated baseline (**Figure 5B**). Increase in ROS formation also showed a CdCl_2_ dose-dependent trend.

### Effects of Overexpressing *Hl-CASP9* on Apoptosis in HEK293T Cells

In order to clarify the functional relevance of *Hl-CASP9* in the mitochondria-dependent apoptosis pathway, pcDNA3.1(+)/*Hl-CASP9* recombinant plasmid (r*Hl-CASP9*) was transfected into HEK293T cells. CCCP was then used to test whether Hl-CASP9 could participate in apoptosis *via* the classical mitochondria-dependent pathway. As noted in **Figure 5C**, the number of cells from the Q3 quadrant (early apoptosis cells) and from the Q2 quadrant (later apoptosis cells) in flow cytometry was calculated. In the absence of CCCP, apoptosis rates were 4.32% and 7.29% in the untransfected control group (HEK293T cells transfected with pcDNA3.1(+) blank plasmid) and transfected control group (HEK293T cells transfected with r*Hl-CASP9*), respectively (**Figure 5C**). The average apoptosis rates of HEK293T cells transfected with r*Hl-CASP9* significantly increased by 1.69 folds (*p* < 0.001) (**Figure 5D**). Upon treatment with CCCP, apoptosis rates in transfected treatment group became 10.92% while that of the untransfected treatment group were only 5.20% (**Figure 5C**). Evidently, apoptosis rate of the transfected treatment group was 2.10-fold (*p* < 0.001) higher than that of the untransfected treatment group (**Figure 5D**), thus implicating overexpressed *Hl-CASP9* as a mediator of mitochondria-dependent apoptosis in HEK293T cells.

### Effects of *Hl-CASP9* Knock-Down in Mitochondrial Stress of Cd-Challenged Coelomocytes

In general, Cd-induced oxidative stress often leads to mitochondrial dysfunction (40). We thus set out to assess any changes in mitochondrial membrane potential and mitochondrial superoxide formation in coelomocytes under Cd challenges. As anticipated, CdCl_2_ (20 μM; 3 h) rapidly induced a loss in mitochondrial membrane, as gauged by the membrane-potential sensitive dye JC-1 (2.5 μM) (**Figures 5E, F**). Similarly, mitochondrial superoxide formation was robustly induced by Cd (20 μM; 3 h) in coelomocytes, as measured by the mitochondrial superoxide probe MitoSOX (2.5 μM) (**Figures 5G, H**). To verify specific effects of Hl-CASP9 on CdCl_2_-treated coelomocytes, experiments of *Hl-CASP9* knock-down by RNAi was carried out. After injection of dsCASP9 for 48 h, the expression of the *Hl-CASP9* mRNA in sea cucumber coelomocytes dropped to 35% in the control group (with no dsRNA) (**Figure S2**). With RNAi by dsCASP9 (48 h), mitochondrial membrane potential was restored in CdCl_2_-treated coelomocytes (**Figures 5E, F**), while formation of mitochondrial superoxide was also lowered (**Figures 5G, H**).

### Effects of *Hl-CASP9* Knock-Down on Coelomocytes Apoptosis

**Figure 6:**
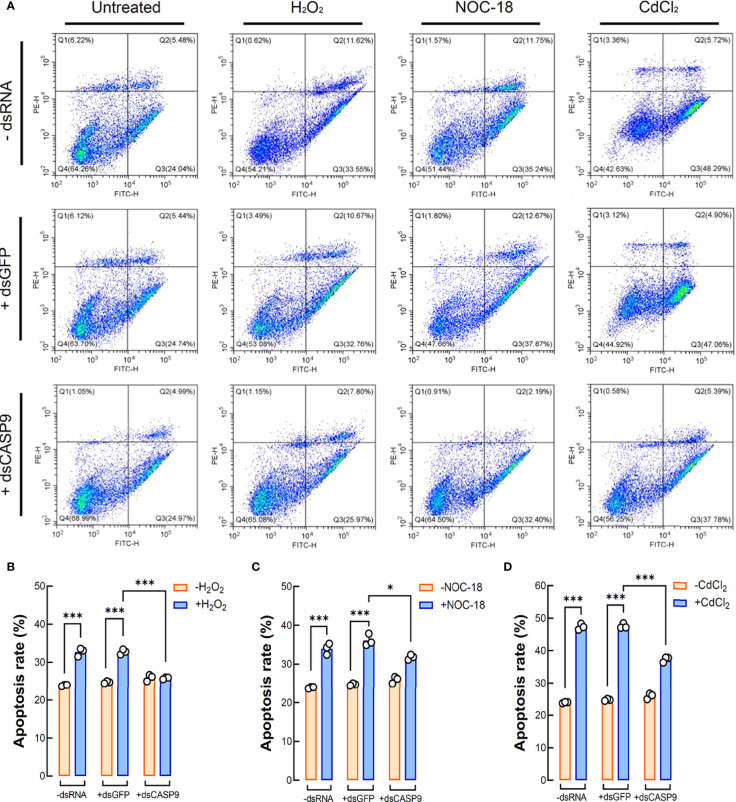
Coelomocyte apoptosis analysis by flow cytometry following *Hl-CASP9* knock-down *in vivo.* **(A)** Coelomocytes treated with the indicated stimulants including H_2_O_2_ (2 μM), NOC-18 (1 μM) or CdCl_2_ (20 μM). Effects are compared among the “-dsRNA” group (sea cucumber injected of RNase-free saline solution), “+dsGFP” group (sea cucumber injected of dsGFP) and “+dsCASP9” group (sea cucumber injected of dsCASP9). **(B)** Statistical analysis on mean rates of early apoptosis in coelomocytes exposed to H_2_O_2_ (2 μM) for 24 h in different groups. **(C)** Statistical analysis on mean rates of early apoptosis in coelomocytes exposed to NOC-18 (1 μM) for 24 h in different groups. **(D)** Statistical analysis on mean rates of early apoptosis in coelomocytes exposed to CdCl_2_ (20 μM) for 24 h in different groups.

Flow cytometry results for coelomocytes apoptosis show that exogenously applied oxidative stress inducers such as H_2_O_2_ (2 μM), NOC-18 (1 μM) and CdCl_2_ (20 μM) could efficiently induce apoptosis in sea cucumber coelomocytes. Early apoptosis rates of coelomocytes under the challenges of H_2_O_2_, NOC-18 and CdCl_2_ in the control group (with no dsRNA) were 32.71%, 33.97% and 47.26%, respectively, whereas corresponding rates in the untreated group was 24.27% (**Figure 6A**). With RNAi by dsCASP9 (48 h), average early apoptosis rates of coelomocytes under these oxidative stress inducers showed a 0.79-fold (*p* < 0.001) (**Figure 6B**), 0.88-fold (*p* < 0.05) (**Figure 6C**) and 0.79-fold (*p* < 0.001) (**Figure 6D**) reduction relative to the negative control group (with dsGFP), respectively. Collectively, these results indicate that coelomocyte apoptosis induced by oxidative stress inducers can be rescued by *Hl-CASP9* knock-down, thus confirming Hl-CASP9 as a direct mediator of such environmental stress cues.

## Discussion

Apoptosis is an intricate process that crucially regulates tissue development, cellular homeostasis and immune defense of the host (26). For their diverse functions in cellular physiology, caspases have been exhaustively investigated in vertebrates, particularly in mammals (14). Several genetic homologues of these mammalian apoptosis effectors have been identified in bivalves, which are evolutionarily ancient invertebrates, hinting that a mitochondria-dependent apoptosis pathway may as well exist in marine invertebrates (41). In mammals, caspase-9 consists of three domains: an *N*-terminal pro-domain, i.e., caspase recruitment domain (CARD), a large catalytic subunit (P20; 20 kDa) and a small catalytic subunit (P10; 10 kDa) (42). Both P20 and P10 subunits are located in the CASc domain, as observed in zebrafish caspase-9 (43). Upon initiation of apoptosis, the P20 and P10 subunits are released and separated from the *N*-terminal region of pro-caspases to form an active heterotetramer (P20-P10)_2_ as an active caspase, which eventually commits irreversibly to apoptosis (44).

To date, the intrinsic mitochondrial apoptosis pathway involved in echinoderm remains scarcely investigated. This present work is the first to identify an echinoderm caspase-9. The deduced amino acid sequence of *Hl-CASP9* contains a conserved caspase-9 architecture encompassing the CARD domain and CASc domain. Hl-CASP9 possesses two active sites rich in histidine and cysteine residues, which is consistent with previous reports that the active site is a catalytic dyad composed of a cysteine sulfohydryl group and a histidine imidazole ring (45). In most caspases, a typical pentapeptide active-site motif “QACRG” can be found, especially in executioner caspases or inflammatory caspases (46). In comparison, in initiator caspases, caspase-8 contains a novel sequence “QACQG” (43), while the pentapeptide “QACGG” is conserved for caspase-9 in vertebrates (37). Intriguingly, *Hl-CASP9* contains an active site “QACRG” located in its P20 subunit. Despite low sequence similarity, evolutionary conserveness of protein structures is observed in caspase-9 and caspase-2 across multiple species from Ambulacraria to higher vertebrates. In multiple sequence alignments of caspase-9 and caspase-2 from human, zebrafish and sea cucumber, a degree of high similarity was revealed in their catalytic domains. *Hl-CASP9* is sequence-wise most akin to the sea cucumber *A. japonicus* caspase-2 and caspase-3/9, suggesting that these caspases are phylogenetically related. As *Hl-CASP9* and the vertebrate caspase-9 are grouped into one clade within the evolutionary tree, we proposed that *Hl-CASP9* is a new member of the caspase-9 family.

It is noteworthy that only a restricted number of caspases (e.g., caspase-9) are amply expressed in the developing nervous system during embryonic development in zebrafish (43). The mitochondria-dependent apoptosis pathway can in turn be activated by multiple mechanisms during diverse processes of embryonic development and cell differentiation (47). In our study, *Hl-CASP9* mRNA showed a dynamic expression pattern at various stages of embryonic and larval development in *H. leucospilota*, implying that *Hl-CASP9* may play some essential roles in apoptosis-regulated embryonic development. Transcript expression of *Hl-CASP9* was detected in all adult tissues examined, with the highest levels being observed in coelomocytes, which are deemed the primary effector cells in the immune system of echinoderms capable of phagocytosis (7, 8). Additionally, *Hl-CASP9* was quite abundantly found in the body wall, which represents a first-line defense against invading microbes such as bacteria. Intuitively, the high mRNA expression levels of *Hl-CASP9* observed in coelomocytes and body wall led us to speculate that *Hl-CASP9* may be an immunity-related gene.

In innate immunity, Toll-like receptors (TLRs) respond to a variety of pathogen-associated molecular patterns (PAMP) and damage-associated molecular patterns (DAMP) to initiate or sustain a coordinated immune response during infection or inflammation. In our previous studies, transcriptional responses of *HLcaspase-6* and *HLcaspase-8* were positively stimulated by LPS or poly(I:C) in *H. leucospilota* coelomocytes (21, 22). To elucidate if a similar response occurs for *Hl-CASP9* under immune challenges, we tested poly(I:C), LPS and heat-inactivated *V. harveyi* as immune stimulants. Surprisingly, *Hl-CASP9* transcript expression was virtually unaltered after these treatments, suggesting that *Hl-CASP9* may not be directly responsible for host responses to infiltrating pathogens. In vertebrates, caspase-9 has been recognized as a core initiator caspase in response to oxidative stress (48). Consequently, we picked H_2_O_2_, NOC-18 and CdCl_2_ as exogenous oxidant donors to test whether *Hl-CASP9* mRNA responds to oxidative stress conditions. H_2_O_2_ is a long-lived major ROS that, in the range of 200-500 μM, can induce apoptosis mediated by the mitochondria-dependent pathway (49). In this study, expression of *Hl-CASP9* in coelomocytes under H_2_O_2_ treatment was dramatically upregulated, in particular at 24 h post-challenge. This result is in agreement with previous studies reporting upregulation of caspase-9 mRNA by H_2_O_2_-induced oxidative stress in BmN-SWU1 cells from the ovary of silkworm and in Schwann cells from the peripheral nervous system of rat (38, 50). In addition to ROS, *Hl-CASP9* transcript expression was also significantly upregulated under treatment with NOC-18, a well-documented ^•^NO donor. When *H. leucospilota* coelomocytes were challenged with CdCl_2_, expression of *Hl-CASP9* mRNA in coelomocytes was strongly elevated, in dose- and time-dependent manners. Previously, enhancement of caspase-9 transcript expression upon Cd challenge was reported in the rat glioma C6 cells (51). In brief, our results show that *H. leucospilota* coelomocytes are sensitive to oxidative stress instigated by H_2_O_2_, NO and Cd, as reflected by *Hl-CASP9* transcriptional changes.

**Figure 7:**
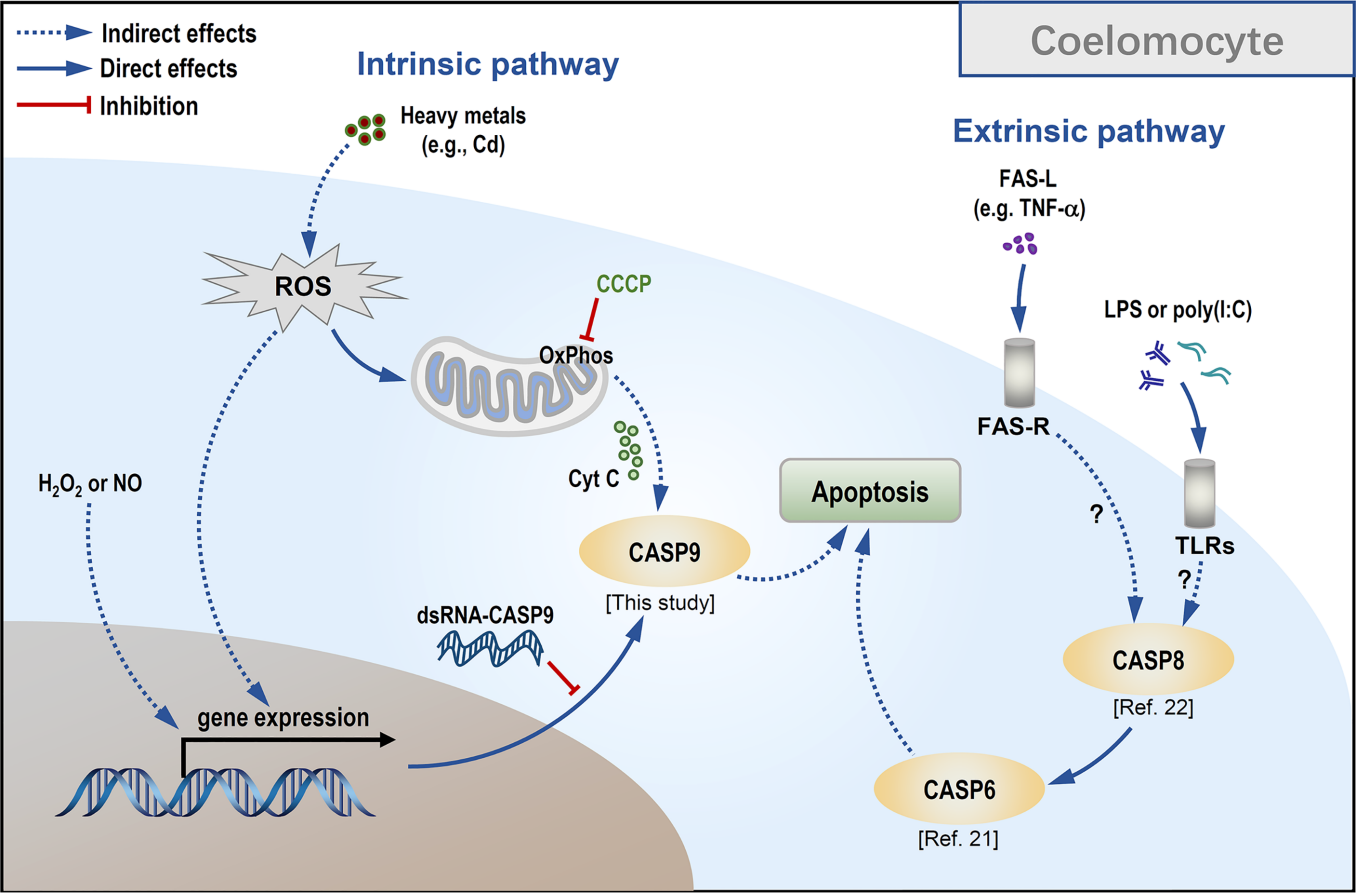
Conceptualization of Cd-induced mitochondria-dependent apoptosis in sea cucumber coelomocytes.

As CCCP is a protonophore capable of uncoupling mitochondrial oxidative phosphorylation leading to a loss of membrane potential on either side of the mitochondrial inner membrane (52), we used it to verify whether Hl-CASP9 participates in Cd-induced apoptosis *via* the mitochondria-dependent pathway. In HEK293T cells overexpressing *Hl-CASP9*, the results corroborate the idea that Hl-CASP9 is a mediator in mitochondria-dependent apoptosis, as in the case of intrinsic apoptosis in mammals (53). As a further test, apoptosis rates of sea cucumber coelomocytes were quantified by flow cytometry following pharmacological treatments with immunostimulants or oxidative stress inducers including Cd. In our previous studies, apoptosis of sea cucumber coelomocytes was significantly upregulated by challenges with LPS or poly(I:C) (22). Here, we showed that H_2_O_2,_ NOC-18 and CdCl_2_ likewise potently induced apoptosis in sea cucumber coelomocytes, suggesting that coelomocytes are intrinsically sensitive to oxidative stress. Our findings support the proposition that oxidative stress can directly activate caspase-9, which has been described in vertebrates (54) and in invertebrates such as freshwater crab *Sinopotamon yangtsekiense* (55) and sea cucumber *S. japonicus* (31). Lastly, results for RNAi experiments show that *Hl-CASP9* knock-down resulted in a significant reduction in coelomocyte apoptosis induced by exogenous oxidative stressors inducing Cd, thus implicating Hl-CASP9 as a direct and crucial mediator of coelomocyte apoptosis in Cd toxicity (See a scheme on concepts in **Figure 7**).

In recent studies, UV irradiation (31), Cd (56) and ZnO nanoparticles (57) were reported to drive oxidative stress by forcing cells to produce excessive oxidants such as H_2_O_2_, ^•^NO, hydroxyl radical (^•^OH), peroxynitrite (ONOO^-^) and hypochlorite (OCl^-^). Unrestrained ROS generation in turn may directly or indirectly alter signal transduction, modulating the outcomes of apoptosis *via* the mitochondria-dependent pathway (58). In the present study, we produced evidence that *Hl-CASP9* upregulation is sufficient to tone up mitochondrial stress in Cd toxicity, by depressing mitochondrial membrane potential and raising mitochondrial superoxide formation.

In summary, an echinoderm caspase-9 was cloned for the first time from the tropical sea cucumber *H. leucospilota* for functional characterization. Our findings give fresh insights into how this ancient caspase-9 mediates coelomocytic apoptosis in response to Cd toxicity *via* a mitochondria-dependent pathway. Evidently, Cd as an important heavy metal in marine pollution can profoundly impact the health and survival of benthic species. There is an urgent need to establish a mechanistic understanding of the organismal and cellular adaptive processes such as autophagy and apoptosis in toxic metal pollution (12). Echinoderms are ancient but useful models to systematically monitor and unveil the impact of environmental perturbations on marine organisms (9). Importantly, bioturbation as a fundamental function of holothurians (i.e., sea cucumbers) within marine benthic systems is intimately linked to the health of coral reefs (59). Our study here has fully demonstrated the utility of *H. leucospilota* as a robust model for the toxicology of heavy metal such as Cd. Further investigation on how Cd impacts processes such as embryogenesis, tissue development, and reproduction in relation to cell death regulation is warranted.

## Data Availability Statement

The original contributions presented in the study are included in the article/**Supplementary Material**. Further inquiries can be directed to the corresponding authors.

## Ethics Statement

Ethical review and approval were not required for the animal study because all animal experiments were conducted in accordance with the guidelines of the South China Sea Institute of Oceanology, Chinese Academy of Sciences.

## Author Contributions

Conception: CR, TC, N-KW. Methodology and investigation: X-ML, XW, ZL, XZ. Resources: XJ, PL, CH, TC, CR. Writing-original draft: X-ML, N-KW. Writing-review and editing: N-KW, CR, X-ML, TC. Supervision and project administration: TC, CR. Funding acquisition: XJ, PL, CH. All authors read and provided inputs on the manuscript

## Funding

This work was graciously supported by grants from the National Key R & D Program of China [2018YFD0901605, 2020YFD0901104], the Key Special Project for Introduced Talents Team of Southern Marine Science and Engineering Guangdong Laboratory (Guangzhou) [GML2019ZD0402], the National Natural Science Foundation of China [42176132, 41906101, 32073002], Li Ka Shing Foundation [510858044 to N.W.] and the Key Deployment Project of Centre for Ocean Mega-Research of Science, Chinese Academy of Sciences [COMS2020Q03].

## Conflict of Interest

The authors declare that the research was conducted in the absence of any commercial or financial relationships that could be construed as a potential conflict of interest.

## Publisher’s Note

All claims expressed in this article are solely those of the authors and do not necessarily represent those of their affiliated organizations, or those of the publisher, the editors and the reviewers. Any product that may be evaluated in this article, or claim that may be made by its manufacturer, is not guaranteed or endorsed by the publisher.
