# Supplementary material for: Evolutionarily Ancient Caspase-9 Sensitizes Immune Effector Coelomocytes to Cadmium-Induced Cell Death in the Sea Cucumber, Holothuria leucospilota
Source: Front Immunol. 2022 Jul 14;13:927880. doi: 10.3389/fimmu.2022.927880 (PMC9330033; doi:10.3389/fimmu.2022.927880)
Supplement: Supplementary file 1 [file DataSheet_1.docx]

# Supplemental Information

**Evolutionarily ancient caspase-9 sensitizes immune effector coelomocytes to cadmium-induced cell death in the sea cucumber, *Holothuria leucospilota***

Xiao-Min Li^1,2,†^, Ting Chen^1,3,4,†^, Xiaofen Wu^6^, Zhuobo Li^1,2^, Xin Zhang^1,2^, Xiao Jiang^1,3,4^, Peng Luo^1,3,4^, Chaoqun Hu^1,3,4^, Nai-Kei Wong^5,*^, Chunhua Ren^1,3,4,*^

|  | **Supplemental Contents** |
| --- | --- |
| **1.** Figure S1. | Sequence analysis of *Hl-CASP9*, related to Figure 1. |
| **2.** Figure S2. | Expression levels of *Hl-CASP9* mRNA in sea cucumber coelomocytes after RNAi, related to Figure 5 and Figure 6. |
| **3.** Table S1. | Primer sequences used in this study. |

# Supplemental Figures

| 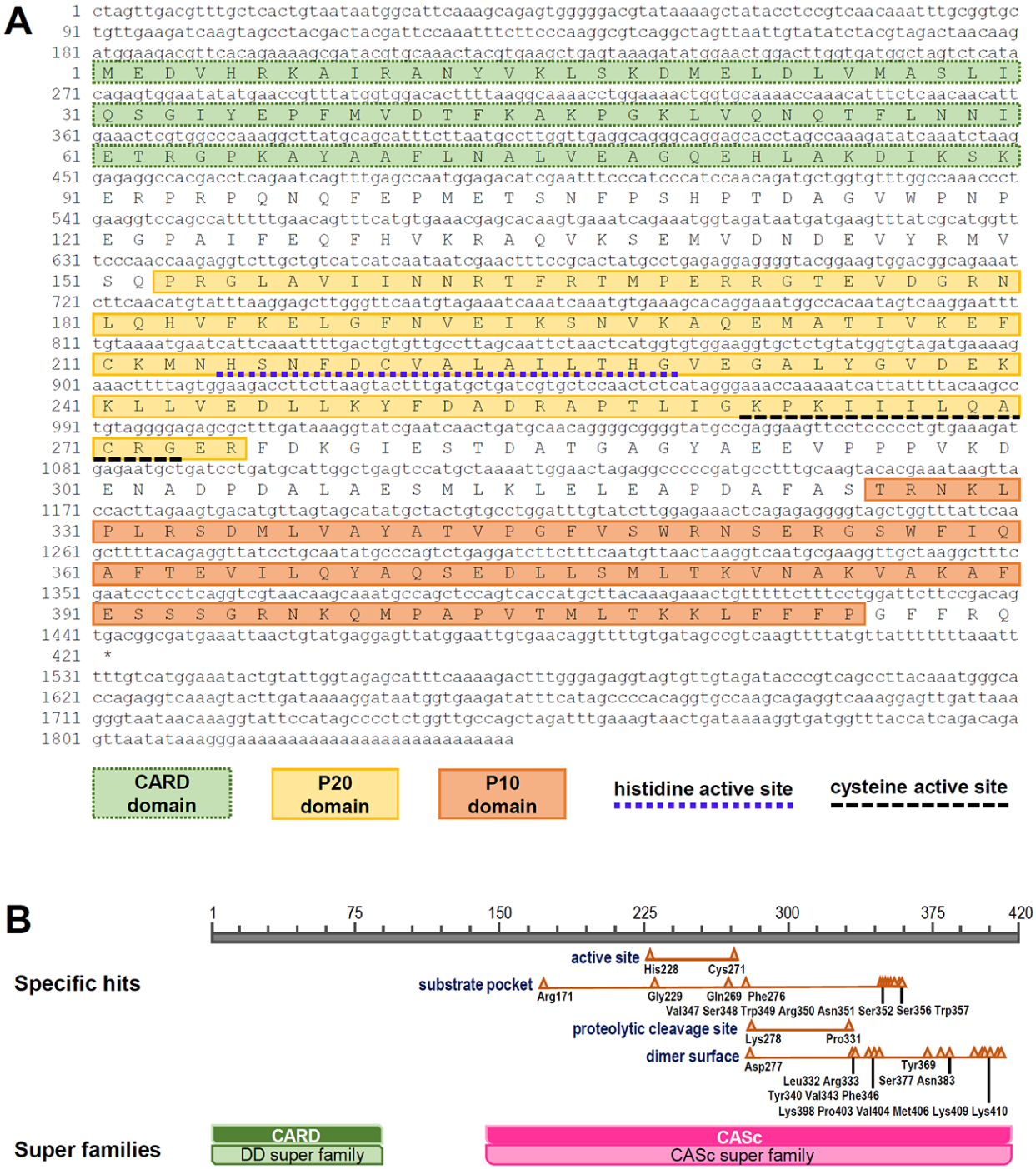 |
| --- |

**Figure S1. Sequence analyses on *Hl-CASP9*, related to Figure 1. (A)** Nucleotide and deduced amino acid sequences of *Hl-CASP9* cDNA. The caspase recruitment domain (CARD), the characteristic caspase subunits P20 and P10, the caspase family histidine active site signature and the cysteine active site motif are marked with different symbols. (**B)** Conserved domains of *Hl-CASP9* were determined by NCBI programs. The active sites, substrate pockets, proteolytic cleavage sites and dimer interfaces are labeled as underlined, and the conserved residues for each domain are marked with a triangle.

|  |
| --- |

**Figure S2. Expression levels of *Hl-CASP9* mRNA in sea cucumber coelomocytes under RNAi, related to Figures 5 and 6.** The groups include: “-dsRNA group” (sea cucumber injected of RFSS), “+dsGFP group” (sea cucumber injected of dsGFP) and “+dsCASP9 group” (sea cucumber injected of dsCASP9).

# Supplemental Table

**Table S1. Primer sequences used in this study.**

| **Name** | **Sequence (5’ - 3’)** |
| --- | --- |
| **For sequence verification** |  |
| *Hl-CASP9*-F | CGGTGCTGTTGAAGATCAAG |
| *Hl-CASP9*-R | TGAACCCAAGCTCCTTAAAT |
| **For cDNA cloning** |  |
| 3’ RACE1 | CATCCCATCCAACAGATGCTGGTG |
| 3’ RACE2  3’ RACE3 | CAAGAGGTCTTGCTGTCATCATC  GAGCTTGGGTTCAATGTAGAAATC |
| 5’ RACE1  5’ RACE2 | CACCAGCATCTGTTGGATGGGAT  TGATTCTGAGGTCGTGGCCTCTCC |
| **For recombinant plasmid construction** |  |
| P*Hl-CASP9*-F  P*Hl-CASP9*-R | CGCGGATCCATGGAAGACGTTCACAGAAAAGCGA  GGTGCTCGACAGTGACAGCCTTCTTAGGTCCTTTC |
| **For qPCR** |  |
| Q*Hl-CASP9-*F  Q*Hl-CASP9-*R  *HLβ-actin*-F  *HLβ-actin*-R  **For dsRNA synthesis**  dsGFP-F  dsGFP-R  dsCASP9-F  dsCASP9-R | GCAAGTACACGAAATAAGTTACCAC  GCTTGAATAAACCAGCTACCC  CCAGAGGAACACCCAGTC  AGGGCGTAACCTTCATAG  GGATCCTAATACGACTCACTATAGGACAAGTTCAGCGTGTCCG  GGATCCTAATACGACTCACTATAGGTTCACCTTGATGCCGTTC  GGATCCTAATACGACTCACTATAGGATGGAAGACGTTCACAGAAAAG  GGATCCTAATACGACTCACTATAGGGCGATAAACTTCATCATTATC |
